# Forecasting egg production in free-range laying hens using multi-farm data

**DOI:** 10.1016/j.psj.2026.107267

**Published:** 2026-06-10

**Authors:** Yusuf Adewale Adejola, Terence Zimazile Sibanda, Isabelle Ruhnke, Johan Boshoff, Mitchell Welch

**Affiliations:** a School of Environmental and Rural Science, University of New England, Armidale, NSW, 2351, Australia; b School of Science and Technology, University of New England, Armidale, NSW, 2351, Australia; c Freie Universität Berlin, Faculty of Veterinary Medicine, Livestock Clinics- Division of Poultry Berlin, Germany; d Computational Analytics Software Informatics, University of New England, Armidale, NSW 2351, Australia

**Keywords:** Egg Production, Free-range systems, Machine learning, Multi-farm data integration, Precision livestock farming

## Abstract

Despite technological advancements in poultry production, accurately forecasting laying rates including sudden drops in egg production remains challenging. This study evaluated the potential of predictive modelling approaches to forecast laying rates and detect reduced production days using data integrated from multiple commercial free-range hen farms. Historical production and weather data from four commercial free-range farms (Farms A–D), comprising 106 flocks and 35,346 flock-days, were analysed. Three Random Forest models were developed: (1) a single-farm model using data from Farm A, (2) a multi-farm model integrating data from Farms B, C, and D, and (3) a combined model incorporating all farms (A–D). Model performance was assessed using Farm A as the target farm. Predictive accuracy was evaluated using the Area Under the Receiver Operating Characteristic Curve (AUC) for classification and Root Mean Squared Error (RMSE) for regression tasks. The single-farm model achieved a median AUC of 0.86 and an RMSE of 2.8, demonstrating strong predictive ability. The multi-farm model achieved a slightly higher AUC (0.89) but lower regression accuracy (RMSE = 4.98). The combined model produced mixed outcomes, improving regression performance (RMSE = 2.55) but resulting in the lowest accuracy (AUC = 0.82). These results demonstrate that models developed using data from one farm can be effectively applied to another, highlighting the potential for cross-farm prediction. Overall, the findings suggest that integrating data from multiple farms can support forecasting of egg production in free-range systems, although combining datasets does not consistently improve model performance. This approach provides a basis for developing practical tools to assist producers in anticipating production changes and improving farm management.

## Introduction

The poultry sector expanded substantially in the number of free-range farming operations due to growing consumer demand for higher welfare standards, particularly in Western nations (Campbell et al., 2020; Miao et al., 2005). However, these systems encounter distinct challenges in sustaining stable laying performance, largely due to variability in environmental conditions and farm management practices (Adejola et al., 2025a). The industry is increasingly adopting smart technologies to improve farm and animal monitoring, allowing quicker and more informed management decisions through the evaluation of large datasets (Sharma and Patil, 2018). Tools such as sensors, cameras, RFID tags, inertial measurement units, motion detectors, and microphones generate substantial amounts of information that contribute to Big Data (Astill et al., 2020). A key challenge, however, is developing effective approaches to analyse these vast datasets to forecast egg production challenges and inconsistent laying performance, particularly within free-range systems.

Machine learning (ML) has emerged as a powerful tool in poultry farming for analysing large datasets generated by IoT-based monitoring systems (Ahmed et al., 2021). These ML techniques provide strong predictive capabilities by using algorithms that adapt and refine their accuracy through continuous data analysis (Pirompud et al., 2024). Several studies have demonstrated the effectiveness of ML in forecasting egg production fluctuations and laying performance. For instance, Morales et al. (2016) applied Support Vector Machines (SVM) to anticipate drops in egg production, achieving an accuracy rate of 98.5%. Similarly, Ramirez-Morales et al. (2017) utilised artificial neural networks (ANN) to identify irregularities in egg production with more than 98.96% accuracy a day in advance. In another study, Gonzalez-Mora et al. (2022) employed Random Forest (RF) models to predict egg production rates, reporting root mean square error (RMSE) values of 0.176% for training and 0.368% for testing when incorporating environmental variables.

Our previous study (Adejola et al., 2025b) built on this foundation and demonstrated the effective application of a Random Forest model for forecasting in free-range systems. Using a 28-day window of on-farm production and environmental data from a single commercial farm was shown to have the best outcome in both classification (identifying problematic production days with an AUC > 0.9) and regression (forecasting laying rates with an RMSE of ∼2.5%). However, this approach was limited to a single farm, leaving uncertainty regarding the generalisability of such models across multiple production systems.

Data integration has become increasingly important in modern agriculture, as reliance on a single data source may limit analytical depth and decision-making capability (Gebresenbet et al., 2023). Integrating multiple streams of data, such as sensor-based data, third-party data sources (e.g., weather and environmental conditions), and historical records, offers a holistic view of farming systems (Gebresenbet et al., 2023). This multi-source approach enables the development of predictive models capable of identifying production trends and unexpected changes, thereby supporting informed decision-making along the value chain (Hashem et al., 2015; Sykuta, 2016). Data sharing among stakeholders also fosters collaboration, addresses shared challenges and collective learning (Schillings et al., 2023).

Despite these advantages, the extent to which models trained on multi-farm datasets can be transferred across production systems remains unclear, particularly in free-range environments characterised by high variability in management practices and environmental conditions. To our knowledge, this study is among the first to integrate multi-farm datasets for forecasting egg production drops and laying rate in free-range hen systems. Therefore, the objective of this study was to evaluate the effectiveness of Random Forest models trained using single-farm, cross-farm, and combined multi-farm datasets for forecasting egg production and detecting sudden production declines. The Random Forest algorithm was retained from our previous study (Adejola et al., 2025b), which focused on single-farm data, to ensure methodological consistency and enable a direct comparison between single-farm and multi-farm modelling approaches. Specifically, this study investigates (i) whether models trained on external farm data can generalise to a target farm, and (ii) whether integrating multi-farm datasets improves predictive performance.

## Material and methods

### Data collection

This study was conducted across four commercial free-range poultry farms (Farms A, B, C, and D), with a total of 106 flocks contributing to the dataset. The participating farms were all commercial free-range egg production systems located in Australia operating under similar industry production standards. The raw datasets comprised production variables including daily egg production per flock, hen age (days), daily mortality rate, feed intake, water intake, and indoor minimum and maximum temperatures. The data collection procedures have been previously described in Adejola et al. (2025b). Flock sizes, housing systems and management practices varied between farms, reflecting the natural variability of the commercial free-range production systems.

Data preprocessing involved the removal of flocks with fewer than 100 days of production records. Missing values were addressed using mean imputation based on simple rolling interpolation. Following cleaning, the final dataset consisted of 35,346 individual daily observations. A detailed breakdown of the dataset is presented in Table 1.

**Table 1:** Breakdown of machine learning datasets.

Farm	Count of Flocks	Total Days
Farm A	7	2772
Farm B	25	10056
Farm C	7	994
Farm D	67	21524
**Total**	**106**	**35346**

Farm A was selected as the case-study farm due to the completeness, consistency, and overall quality of its dataset. In comparison with Farms B–D, Farm A exhibited fewer missing values (<1%), continuous daily records with minimal reporting gaps, and comprehensive coverage of all key production and environmental variables. These attributes made it particularly suitable for model benchmarking and validation.

Data from Farm A were stored within a relational database system, with daily synchronisation from on-farm data collection systems to a central database. This ensured that recorded values accurately reflected daily observations with minimal missing values (<1%), and no carry-over entries to compensate for missed reporting days. Any missing data points were addressed using localised median imputation, where any missing values were replaced using rolling medians calculated within a ± 3-day window surrounding each missing data point, rather than global dataset medians. The Farm A dataset comprised 2,772 days of data, representing the full production cycle (18–74 weeks of age) across seven flocks.

In contrast, Farms B–D relied on manual and semi-automated reporting systems, resulting in slightly lower data consistency. Farms B–D followed the same production cycle (18–74 weeks of age), with missing data accounting for less than 5% of observations, these gaps were addressed using localised median imputation, where any missing values were replaced using rolling medians calculated using the same approach used for farm A for consistency.

Table 2 summarises the daily production variables alongside climatic data obtained from the Bureau of Meteorology, corresponding to the geographical location of all the farms. The combined dataset incorporates key production variables, consistent with those utilised in previous studies (Morales et al., 2016; Omomule et al., 2020), as well as climatic and environmental indicators examined by Gonzalez-Mora et al. (2022). This alignment ensures methodological consistency with established research in the field. To enhance interpretability, the dataset was further refined by transforming variables into their mean and standard deviation representations. This feature reduction approach retained only those variables that consistently demonstrated high feature-importance rankings in our previous study (Adejola et al., 2025b).

**Table 2:** Raw production and location-specific climate data for all Farms.

Data Item	Unit.
Laying Rate	%
Mortality	%
Feed intake/hen/day	g
Water intake/hen/day	ml
Indoor Minimum Temperature	°C
Indoor Maximum Temperature	°C
Relative Humidity 9 am	%
Relative Humidity 3 pm	%
Outdoor Maximum Temperature	°C
Outdoor Minimum Temperature	°C
Precipitation	mm/day
Solar Radiation	MJ/m^2^/day

### Feature Dataset

Feature engineering was applied to transform raw time series data into more informative features to improve performance of the machine learning models. This strategy enhances the model’s ability to capture relevant patterns in the data, leading to more accurate predictions (Čistý et al., 2024). Features were extracted over a moving window, enabling the model to capture underlying patterns and trends in the data while minimising the impact of noise. Following findings from our previous work (Adejola et al., 2025b), a 28-day sliding window approach was chosen as it showed to have the best performance for the regression task trained on the models. For each 28-day window across each flock, the following aggregate features were calculated for each of the raw variable. These consisted of:

• Mean
• Standard Deviation

In addition to the window features, two additional items are included as features:

• The age of the flock at the start of the data window.
• The difference between the population average for the age of the hens and the production rate on the last day of the 28-day data window. This indicates the relative performance of the flock at that point in its production life compared to the expected average for the population.

This process produced a single modelling dataset using a 28-day rolling window, such that each forecast was based on data from the 28 consecutive days immediately preceding the forecast date for each flock. The selection of these features was informed by the findings from Adejola et al. (2025b) where a Random Forest model trained on a 28-day window of production and environmental data demonstrated that these features consistently ranked highly in feature importance analyses, underscoring their predictive value. Based upon the overall raw data available, features were included for the laying rate, mortality rate, indoor minimum temperature and indoor maximum temperature for the forecasts on the case study flocks. After all data cleaning, removal of incomplete flocks and unusable days, the overall training dataset contained 9701 data points with a further 2505 data points in the case study farm (totalling 12206 data points).

### Targets for forecast

Two forecast targets were derived, mirroring the approach of study by Adejola et al. (2025b):

• The laying rate
• Laying rate fluctuations (i.e. sudden drops in the production rate)

For the regression task, the target variable was the laying rate of each flock, representing a continuous measure of production performance. In previous studies, the raw laying rate had been commonly employed as the target variable for forecasting production outcomes (Gonzalez-Mora et al., 2022; Omomule et al., 2020). However, a major limitation of using the raw laying rate as the target variable in regression task is its sensitivity to sudden fluctuations. For the machine learning model to generalise, it may have a reduced sensitivity to outlier values such as those seen in sudden reductions related to problematic production fluctuations (i.e. the machine learning algorithm will likely fit to the general trends in a production curve without capturing the sudden fluctuations). Nevertheless, features derived from the laying rate have been shown to be useful in earlier studies (Morales et al., 2016), who found that previous laying performance is a strong predictor of future output. A second binary parameter for forecast is proposed that specifically targets the problematic fluctuations creating a classification task. This parameter simply flags a production day as problematic (1) or normal (0), resulting in a simple binary classification scenario. To construct this classification target, a prominence-based detection algorithm was applied to the raw laying rate time-series. This reflects the approach established in Adejola et al., 2025b. In summary, the algorithm implements a rolling window approach as used that identifies local minimums in the data by comparing each data point to the average from a proceeding 7-day window of data. The purpose of this 7-day window is solely to establish the actual occurrence of a production drop as they are typically measured over a week. The local minimums in the curve with the 7-day window, which represent sudden drops in egg production, were then included in the final set based upon the measure of ***prominence*** and ***duration***. The minimum prominence of 1.5% and 2.5% were selected in this study to identify the local minimums that is days when the egg production drops substantially due to the broader farm-based model across all datasets. The small target identified days when the laying rate decreased by at least 1.5% relative to the preceding 7-day window, whereas the large target identified days with decreases of 2.5% or greater. This decision is informed by the sensitivity analysis in the previous works (Adejola et al., 2025a, 2025b). The duration was defined as the number of data points that comprised the production drop within 3 days.

**Fig. 1:**
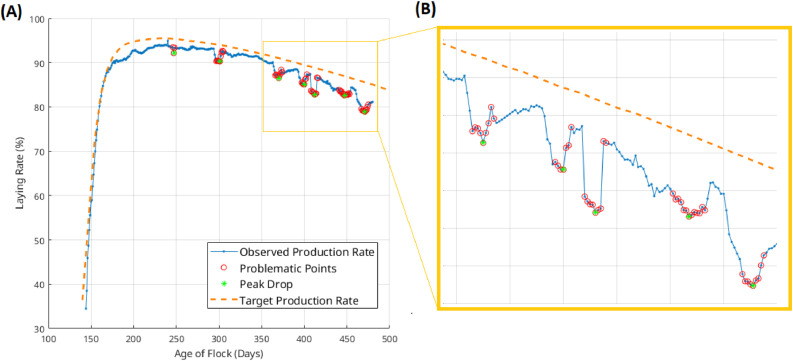
(A) An Example of a production egg curve from the dataset under study illustrating the identification of problematic regions using the prominence-based detection algorithm (Adejola et al., 2025a). The orange line plots the target production rate for the Lohamnn Brown laying hens as a reference that illustrates an ideal production scenario. The inset (B) shows the identification of the problematic days that form the sudden production fluctuations.

Any point that lied within one of these production fluctuations was identified as a target for the algorithm to forecast. In this study a single target set with generated using minimum prominence of 1.5, resulting in a set with 1009 problematic days was being identified across the entire dataset and 435 in the training set from the case study farm (A). Fig. 1 plots and exemplar production curve with the sudden fluctuations in production identified by the algorithm.

### Case study structure

The case study was broken down into the three individual experiments to address the research questions regarding the use of machine learning workflows for forecasting production outcomes.

**Fig. 2:**
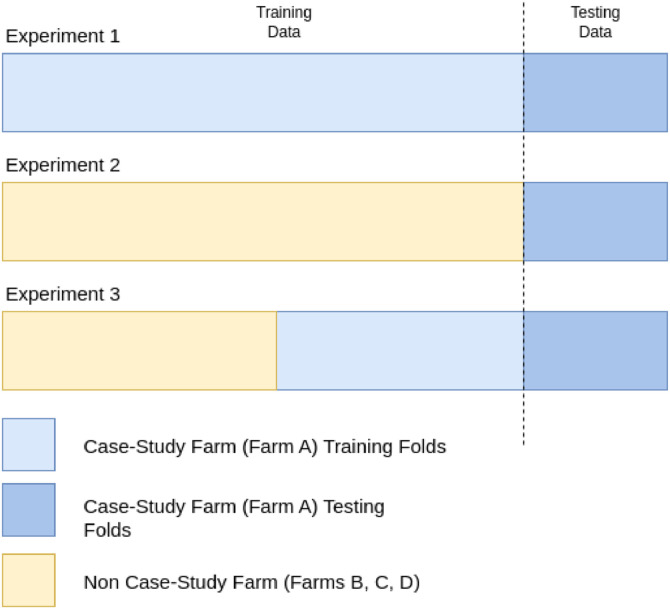
Structure of the datasets and experimental design. In Experiment 1, only Farm A data were used, with each of its seven flocks serving once as the testing flock (dark blue) while the remaining six flocks were used for training (light blue). In Experiment 2, data from Farms B–D (yellow)were used for training, and all Farm A flocks were used for testing. In Experiment 3, data from all farms (A–D) were combined for training, with each flock from Farm A again used independently as a testing flock. Error bars represent variation in the number of data points per farm.

The schematic in Fig. 2 provides and outline of the data The first experiment takes data from *only* the case study farm (A). The dataset was systematically divided into the 7 flocks that comprised the data, with each flock used as a fold (or division) for testing and training of a machine learning model. In this arrangement, each flock served as a testing flock, with the remaining 6 flocks being used for training the model. All flocks were tested in this way to understand the forecasting performance across each individual flock. This experiment provided an example of a real-world use case where the producer uses historical datasets from their own farm to train a system for forecasting performance on other future flocks.

The second experiment used data that was obtained only from the extended datasets (Farm B, C, D)**,** excluding the data from the case study (Farm A) in the training set but used the flocks from the case study farm A as the testing dataset. In this arrangement, each flock from the case study farm A served as a testing flock (all 7 flocks). This experiment provided an example of a real-world use case where the producer uses a larger database including historical datasets from other producers to train a system for comparison forecasting performance on their farm.

The third experiment takes a combined dataset that includes data from the case study farm and from the wider dataset of all farms. This experiment provides an example of a real-world use case where the producer uses a historical dataset from other producers augmented by their own historical dataset to train a system for forecasting performance on future flocks on their farm. This represents the most ideal situation where the case study farm can benefit from (anonymised) data that is shared from other producers to improve the forecasts without relying solely on their own historical data. It also helps address the scenario where there is some historical data is available for a particular producer, but it is insufficient to effectively train a machine learning model.

To ensure there was no overlap between training and testing datasets, all experiments were designed so that testing flocks were completely excluded from the training process preventing an unbiased estimate of model performance.

### Machine learning workflow

A machine learning workflow using the random forest algorithm was implemented and applied to each of the datasets outlined in the previous section and trained on the classification (both large and small targets) and regression targets for a forecasting interval of 1-day. The results from the machine learning study presented by Adejola et al. (2025b) were used to select an optimal configuration for the hyperparameters for the random forest. The base dataset was initially divided for each experiment with the non-testing flock further divided into randomised stratified training/validation and testing sets comprising of 75% and 25% of the data respectively. The algorithm was initially trained using the training/validation set used to tune the parameters of the algorithm (i.e. the validation step). The test flock was then used provide a robust out-of-sample performance measure of the final trained algorithm. All input data features were z-score normalised so that they had a mean of 0 and a standard deviation of 1 to maximise performance across data features that represent different measures and ranges. The data was balanced using random under-sampling of majority class (0 in this case) the so that the algorithm was ultimately trained on a balanced set to avoid biasing the algorithm. A random forest algorithm was trained and optimised by tuning the number of decision trees used in the ensemble using the training and validation datasets. This reflects the approach adopted by Adejola et al. (2025b). The performance of the trained algorithm was assessed using the out-of-sample testing set. The workflow for the regression analyses was very similar, except the initial random sample did not require any stratification and the balancing step was removed as there were no classes the balance in the regression problem. For each of the individual experiments, 1000 trials were completed with a full set of performance metrics (outlined in the next section) for each experiment. This provides a distribution of the expected performance and an indication of the robustness. MATLAB R2023a (MathWorks, 2023) was used to implement the machine learning workflow and experimental trials were completed using a 64-core Intel Xeon Skylake CPU with 320 GB of Memory.

### Performance Metrics

The *Area Under the Receiver Operating Characteristic Curve* (AUC-ROC) is a metric for evaluating the performance of classification models. The ROC curve provides a graphical illustration of a model’s capacity to differentiate between classes across various decision thresholds. The AUC measures the total area beneath this curve, representing the model’s overall discriminative ability between positive and negative cases. Higher AUC values, approaching 1.0, denote stronger classification performance and a greater ability to correctly rank observations according to different classes. An AUC value of 0.5 or below represents poor performance that is equal to random chance.

For the regression analysis, the *Root Mean Squared Error* (RMSE) was employed as the evaluation metric. RMSE quantifies the average magnitude of deviation between the predicted and observed values, providing an overall measure of model accuracy. It is calculated as the square root of the mean of the squared differences between predicted and actual outcomes, offering an interpretable measure of prediction error in the same units as the dependent variable.

(i) $$RMSE=\;\sqrt{\frac{\sum_{i=1}^{n}\left(y_{i}-\;{\hat{y}}_{i}\right)}{n}}$$

## Results

**Fig. 3:**
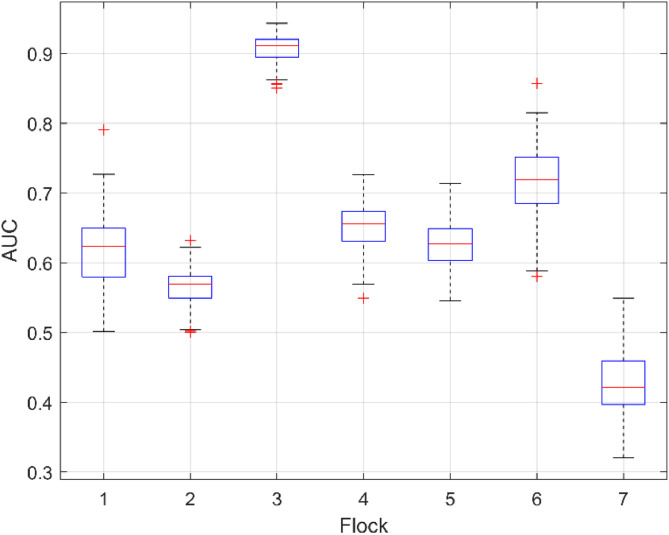
The performance distributions consisting of the Area-under-curve values for all case study flocks from Farm A (experiment 1).

**Fig. 4:**
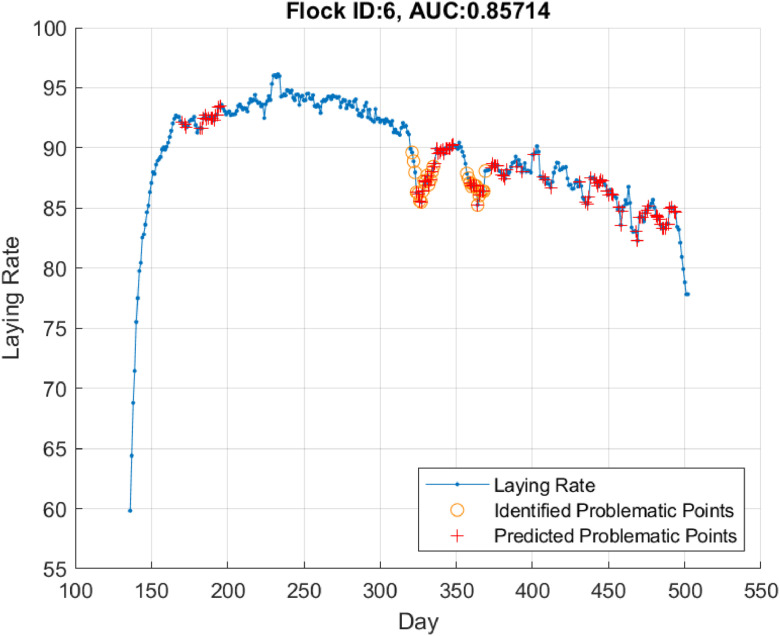
An exemplar laying-rate production curve for flock 6 from the case study farm A used during experiment 1 (classification problem based upon case study farm only dataset), plotting the observed laying rate (blue ‘.’), the identified problematic points (orange circles') and the forecasted problematic days (red crosses). The reported model performance for across this flock is ∼ 0.857 AUC. **Laying rate values are expressed as percentages (%).**

**Fig. 5:**
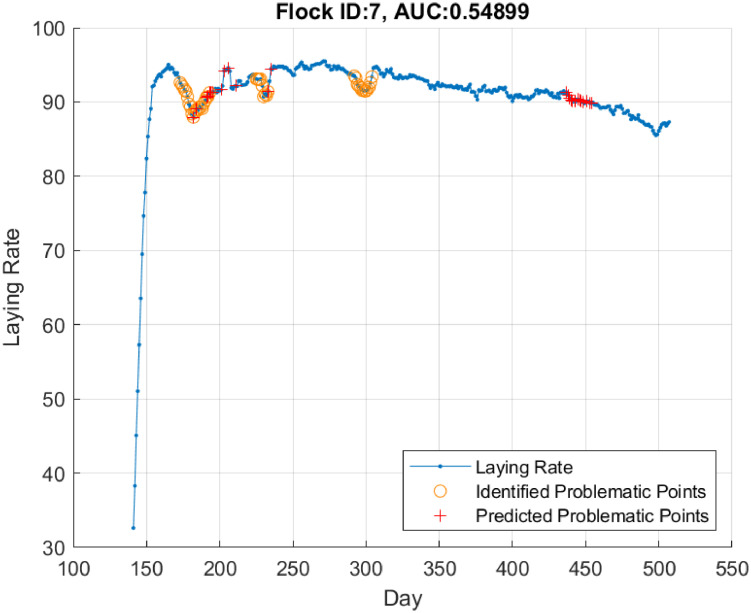
An exemplar laying-rate production curve for flock 7 from the case study farm A used during experiment 1 (classification problem based upon case study farm only dataset), plotting the observed laying rate (blue ‘.’), the identified problematic points (orange circles') and the forecasted problematic days (red crosses). The reported model performance for across this flock is ∼ 0.55 AUC. **Laying rate values are expressed as percentages (%).**

**Fig. 6:**
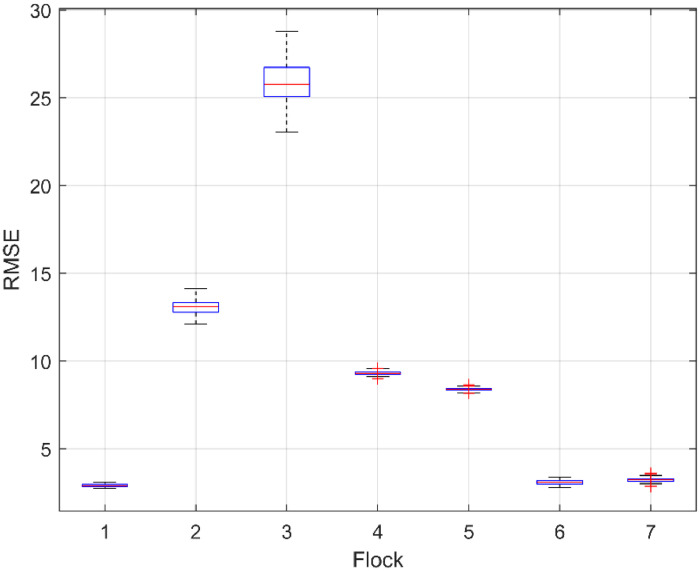
The performance distributions consisting of the RMSE values for all case study flocks for experiment 1 (regression problem based upon case study farm only dataset).

**Fig. 7:**
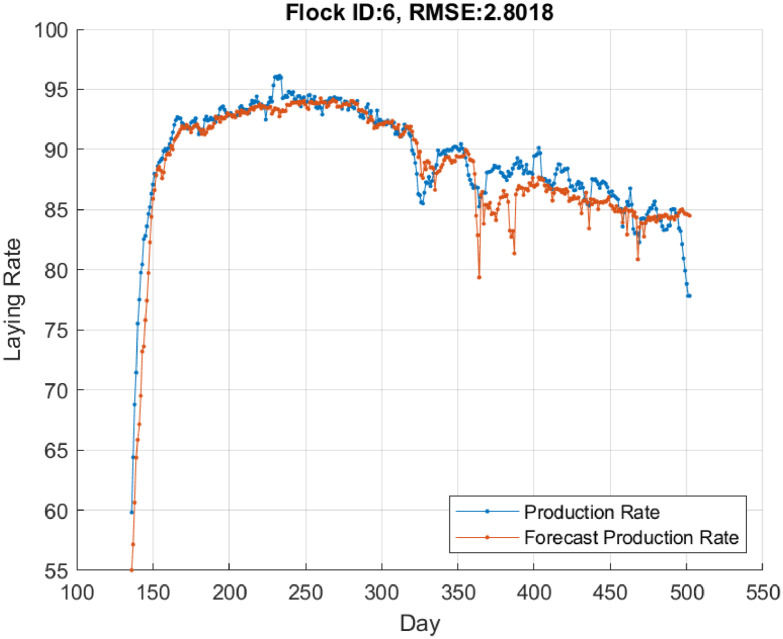
An exemplar laying-rate production curve for flock 6 from Farm A for experiment 1 (regression problem based upon case study farm only dataset), plotting the observed laying rate (blue lines and dots‘.’), the forecasted laying rate (red lines and dots '.'). The reported model performance for this flock was ∼2.8 RMSE. **Laying rate values are expressed as percentages (%).**

**Fig. 8:**
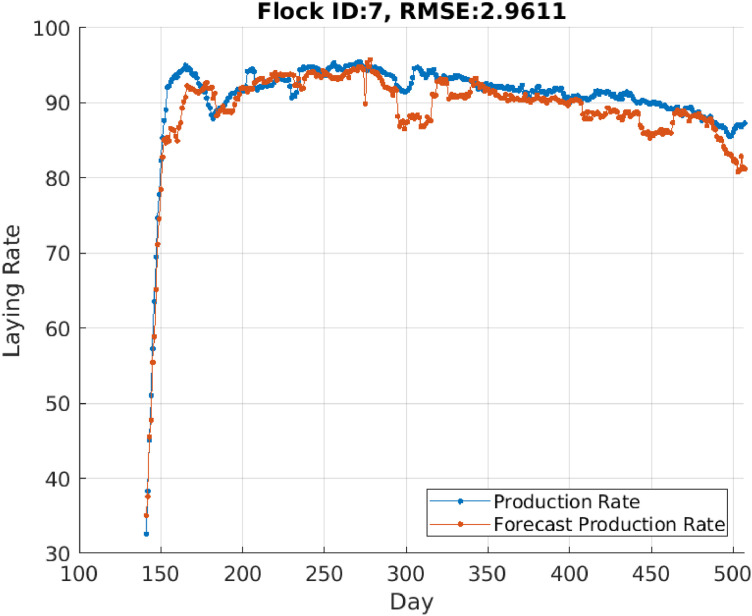
An exemplar laying-rate production curve for flock 7 from Farm A for experiment 1 (regression problem based upon case study farm only dataset), plotting the observed laying rate (blue lines and dots‘.’), the forecasted laying rate (red lines and dots '.'). The reported model performance for this flock was ∼2.96 RMSE. **Laying rate values are expressed as percentages (%).**

The results from experiment 1 are presented in Figs. 3–8 for both the classification task (Figs. 3, 4& 5) and regression task (Figs. 6, 7& 8). Recall experiment 1 used the data sourced only from the farm A. Fig. 3 presents the AUC values for forecasting the problematic production days across trials on the 7 flocks from the case study farm. The median AUC values vary significantly across the flocks, indicating that there is a significant level of variation introduced by production differences between flocks (e.g. different sheds, different management approaches, etc.) even within just the case study farm. The results indicate that most flocks achieve an AUC greater than 0.6 indicating a reasonable fit of the model for the classification task.

Fig. 4 presents an example of overall higher performing flock (flock 6) from Farm A obtained during experiment 1. The target points, consisting of the identified problematic days are plotted on the curve using orange markers. The forecast results are plotted using red crosses. It is evident that there is significant overlap between the forecast and target points, with the two major production drops in the centre of the curve correctly identified. Most of the incorrectly forecasted points occur in areas of the production curve where there is larger variability in the laying rate (standard deviation exceeds 2.5% of the mean laying rate). In contrast, Fig. 5 presents an example of overall lower performing flock (Flock 7) from Experiment 1, which achieved an AUC of 0.55. Although the model correctly identified some problematic production periods, the overlap between the predicted problematic points (red crosses) and the actual problematic points (orange circles) was considerably lower than that observed for the best-performing flocks.

Fig. 6 plots the distributions of the RMSE values for the regression analysis, demonstrating the effective fit for the regression model. This is also demonstrated in the exemplar production curve in Fig. 7, which plots the forecast values (red) and target values (blue). The forecast values follow the general trend of the targets, capturing the key features of the curve. This indicates that the feature set provides good predictive performance for forecasting the laying rate. In contrast to the exemplar high-performing flock, Fig. 8 presents the regression results for Flock 7, which represented an example of overall lower performing flock in Experiment 1. The model was still able to capture the overall production trajectory throughout the laying cycle.

**Fig. 9:**
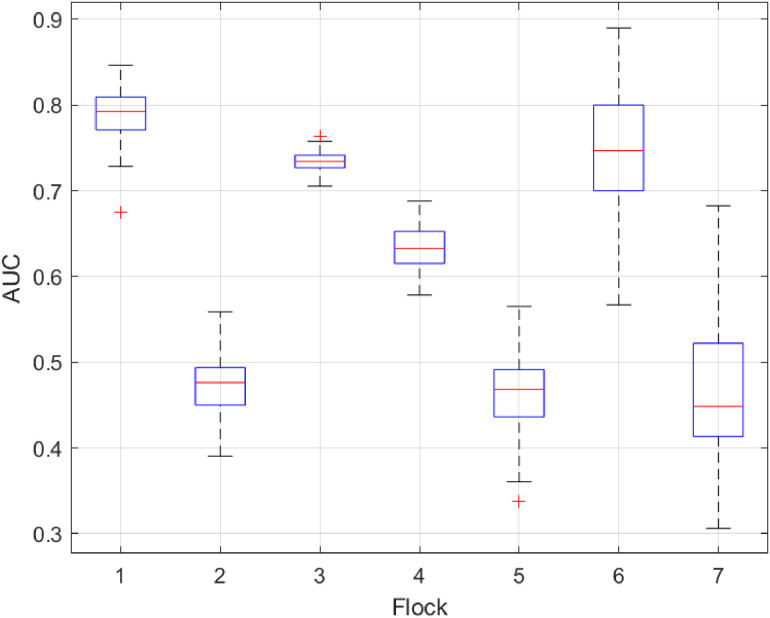
The performance distributions consisting of the Area-under-curve values for all case study flocks (obtained from Farm B-D) for experiment #2 (classification problem based upon non-case study farm dataset).

**Fig. 10:**
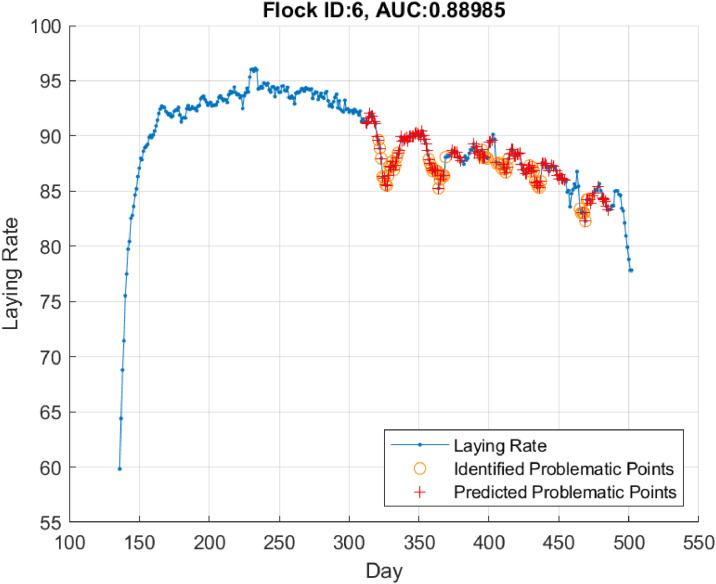
An exemplar laying-rate production for flock 6 from the case study farm for experiment 2 (classification problem based upon training using only non-case study farm datasets (Farm B-D)), plotting the observed laying rate (blue lines and dots ‘.’), the identified problematic points (orange circles) and the forecasted problematic days (red '+'). The reported accuracy for across this flock was ∼ 0.89 AUC. **Laying rate values are expressed as percentages (%).**

**Fig. 11:**
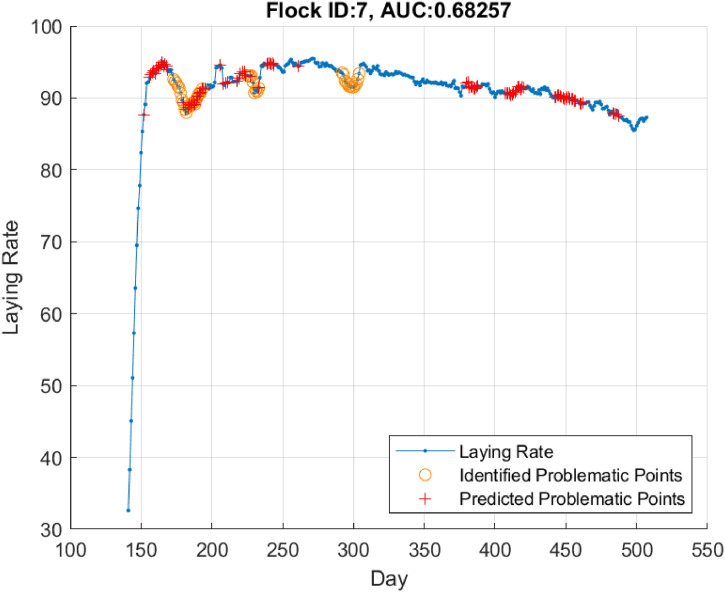
An exemplar laying-rate production for flock 7 from the case study farm for experiment 2 (classification problem based upon training using only non-case study farm datasets (Farm B-D)), plotting the observed laying rate (blue lines and dots ‘.’), the identified problematic points (orange circles) and the forecasted problematic days (red '+'). The reported accuracy for across this flock was ∼ 0.68 AUC. **Laying rate values are expressed as percentages (%).**

**Fig. 12:**
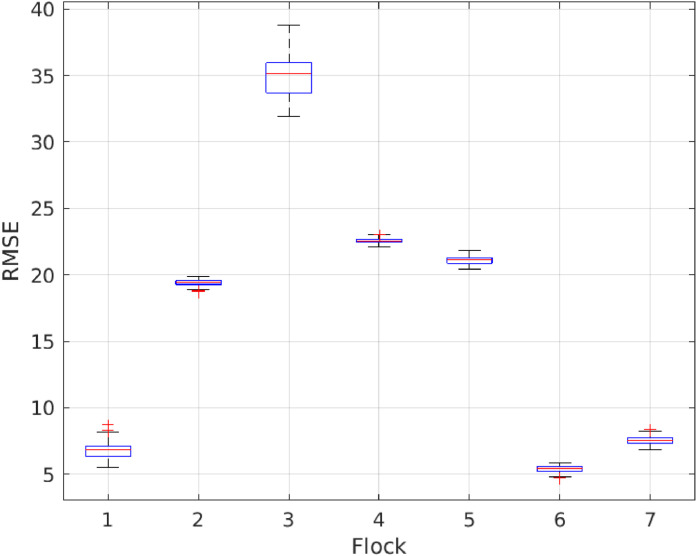
The performance distributions consisting of the RMSE values for all 7 case study flocks obtained from Farm A using for experiment 2 (regression problem based upon non-case study farm only dataset).

Results for the second experiment are plotted in Fig. 9, Fig. 10, Fig. 11, Fig. 12. Fig. 9 presents the AUC values for forecasting the problematic production days across trials on the 7 flocks from the case study Farm A tested using models trained exclusively on data from Farms B, C, and D as experiment 1, the median AUC values varied across the flocks, indicating that there was a substantial level of variation introduced by production differences between flocks (e.g. in this case different producers, different management approaches etc.)

Fig. 10 plots an exemplar production curve (laying rate) for flock 6, again, representing an overall higher performing flock from the case study farm A obtained during experiment 2. The target points, consisting of the identified problematic days are plotted on the curve using orange markers. The forecast results are plotted using red crosses. There was substantial overlap between the forecast and target points, with the two major production drops in the centre of the curve. The results demonstrate a higher level of performance which denotes improved predictive accuracy compared to the one observed in experiment 1 with an AUC of approximately 0.89 As before, most of the incorrectly forecast points occur in areas of the production curve where there is larger variability in the laying rate. Fig. 11 presents an exemplar of overall lower performing flock (Flock 7) from Experiment 2, which achieved an AUC of 0.68. Compared with the highest-performing flock, the model exhibited reduced ability to accurately discriminate between problematic and normal production days.

**Fig. 13:**
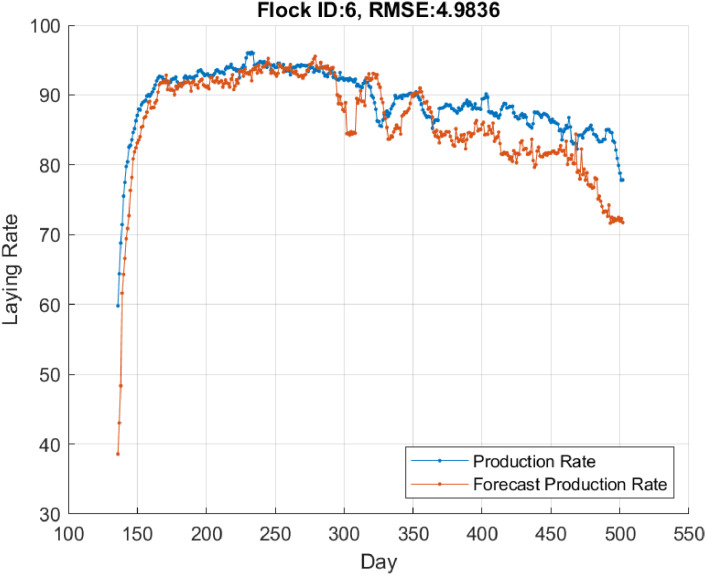
An exemplar laying-rate production curve for flock 6 from the case study farm A for experiment 2 (regression problem based upon only non-case study farm dataset), plotting the observed laying rate (blue ‘.’), the forecasted laying rate (red '.'). The reported performance for this flock was ∼4.98 RMSE. **Laying rate values are expressed as percentages (%).**

**Fig. 14:**
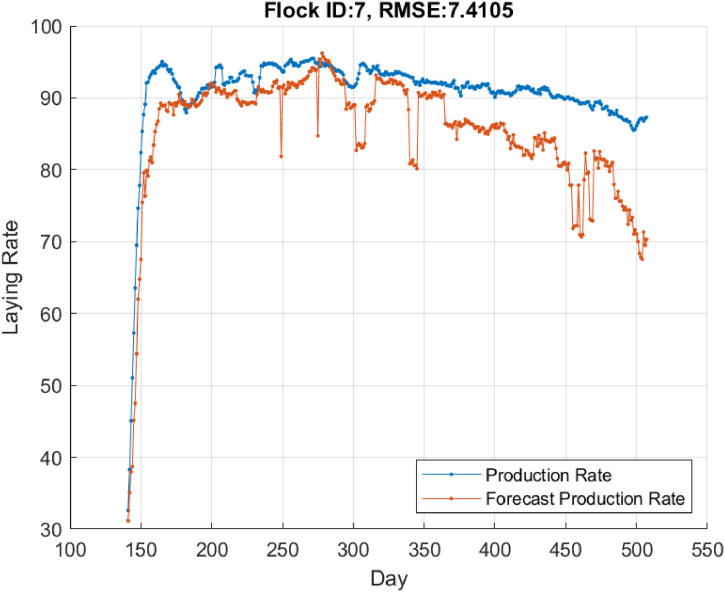
An exemplar laying-rate production curve for flock 7 from the case study farm A for experiment 2 (regression problem based upon only non-case study farm dataset), plotting the observed laying rate (blue ‘.’), the forecasted laying rate (red '.'). The reported performance for this flock was ∼7.41 RMSE. **Laying rate values are expressed as percentages (%).**

Fig. 12 plots the distributions of the RMSE values for the regression accuracy, demonstrating an effective fit for the regression model with slightly lower performance than that demonstrated in experiment 1. This is also demonstrated in the exemplar production curve in Fig. 13, which plots the forecast values (red) and target values (blue). We can see that the forecast values follow the general trend of the targets, capturing the key features of the curve. The higher RMSE of ∼4.9 indicates lower performance than that demonstrated on the regression task in experiment 1 (Fig. 6). This indicates the feature set provides good predictive performance for forecasting the laying rate; however, the predictions are not as reliable when data from the case study farm is not used. Fig. 14 presents the regression results for Flock 7, which represented the example of overall lower performing flock in Experiment 2. The model achieved an RMSE of 7.41, indicating a considerably larger prediction error than that observed in the best-performing flocks and in the corresponding lowest-performing flock from Experiment 1. Although the forecasted laying rates broadly captured the initial increase in production and the timing of peak lay, prediction accuracy progressively deteriorated as the flock aged.

**Fig. 15:**
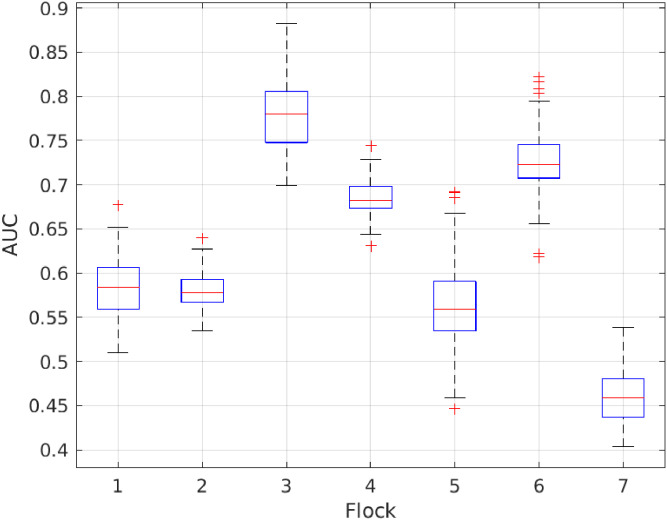
The performance distributions consisting of the Area-under-curve values for all case study flocks for experiment #3 (classification problem based upon combined dataset).

**Fig. 16:**
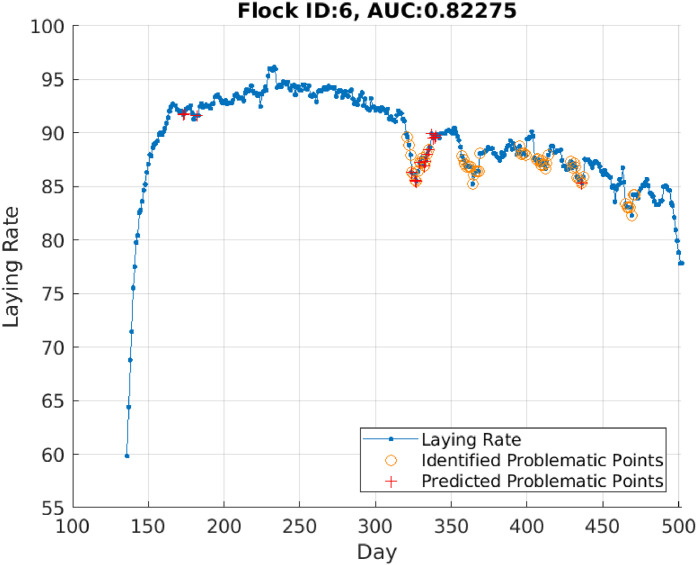
An exemplar laying-rate production for flock #6 from the case study farm for experiment #3 (classification problem based upon combined dataset), plotting the observed laying rate (blue ‘.’), the identified problematic points (orange 'o') and the forecasted problematic days (red '+'). The reported performance for across this flock is ∼ 0.82 AUC. **Laying rate values are expressed as percentages (%).**

**Fig. 17:**
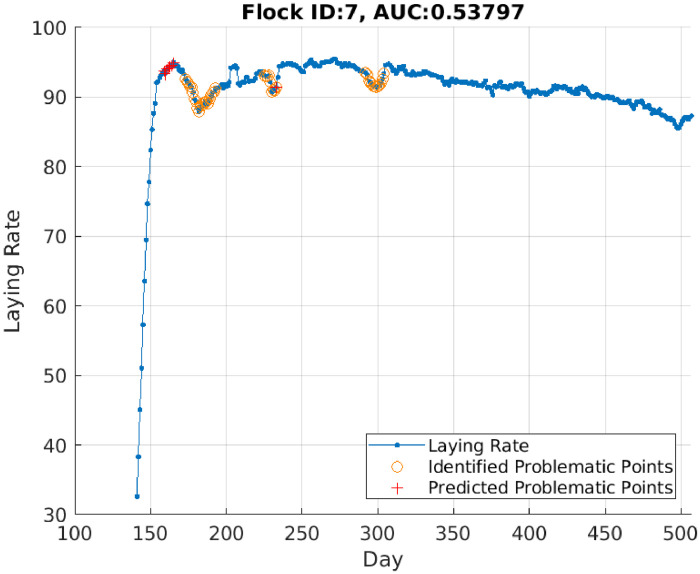
An exemplar laying-rate production for flock #7 from the case study farm for experiment #3 (classification problem based upon combined dataset), plotting the observed laying rate (blue ‘.’), the identified problematic points (orange 'o') and the forecasted problematic days (red '+'). The reported performance for across this flock is ∼ 0.54 AUC. **Laying rate values are expressed as percentages (%).**

**Fig. 18:**
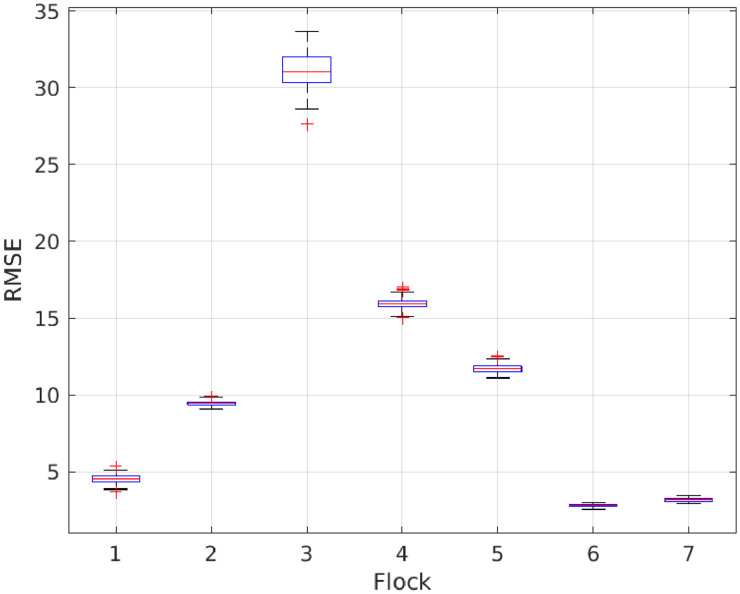
The performance distributions consisting of the RMSE values for all case study flocks for experiment #3 (regression problem based upon combined dataset).

**Fig. 19:**
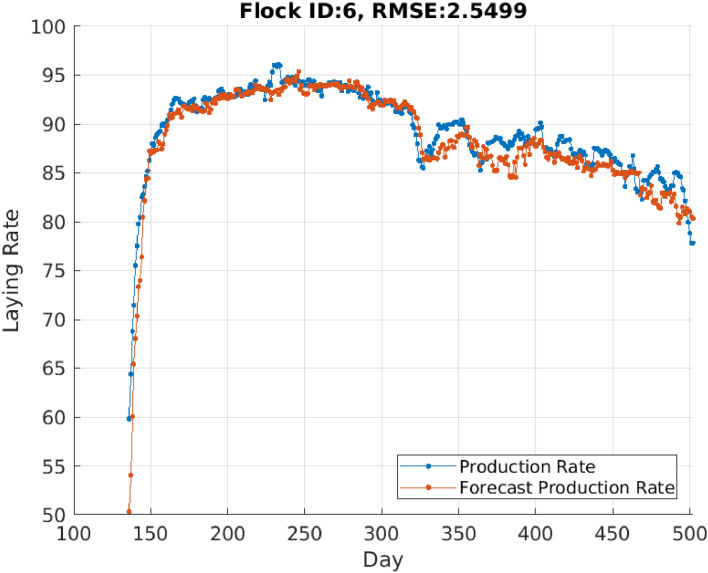
An exemplar laying-rate production curve for flock #6 from the case study farm for experiment 3 (regression problem based upon combined dataset), plotting the observed laying rate (blue ‘.’), the forecasted laying rate (red '.'). The reported performance for this flock was ∼2.55 RMSE. **Laying rate values are expressed as percentages (%).**

**Fig. 20:**
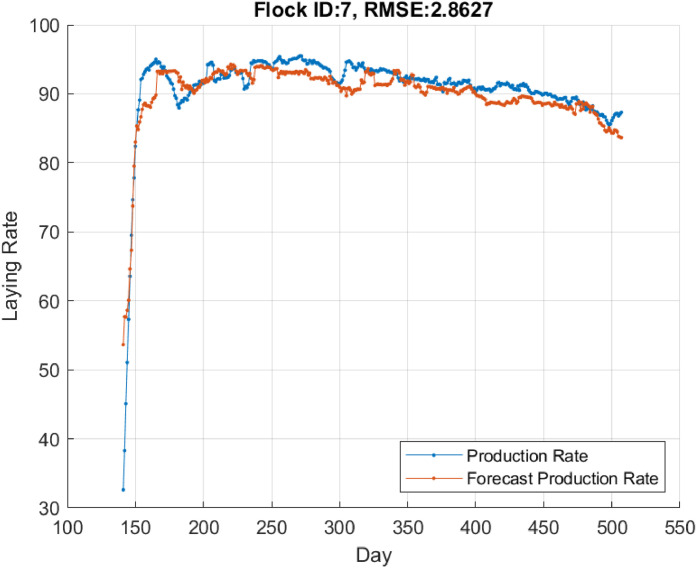
An exemplar laying-rate production curve for flock #7 from the case study farm for experiment 3 (regression problem based upon combined dataset), plotting the observed laying rate (blue ‘.’), the forecasted laying rate (red '.'). The reported performance for this flock was ∼2.86 RMSE. **Laying rate values are expressed as percentages (%).**

Results for the third experiment are plotted in Fig. 15, Fig. 16, Fig. 17, Fig. 18, Fig. 19, Fig. 20. Fig. 15 presents the AUC values for forecasting the problematic production days across trials on the 7 flocks from farm A using the models trained on the combined dataset consisting of data from the case study farm and data sourced from the other producers. As with experiments 1 and 2, the median AUC values vary substantially across the flocks, indicating that there was a substantial level of variation introduced by production differences between flocks (e.g. in this case different producers, different sheds, different management approaches etc.)

Fig. 16 plots an exemplar production curve (laying rate) for flock 6, representing an overall higher performing flock from the case study farm. The target points, consisting of the identified problematic days are plotted on the curve using orange markers. The forecast results are plotted using red crosses. It is evident that there is significant overlap between the forecast and target points however many of the problematic days have not been detected in the flock. This is further evident with the lower AUC value (0.82) achieved for this test flock. Overall, this demonstrates that the combined dataset produces a model that is less sensitive to the problematic points, producing a larger number of false negatives in its predictions. The overall lower-performing flock (flock 7) example for Experiment 3 (Fig. 17) further illustrates the limitations of the combined dataset approach for classification tasks. In this example, the model achieved an AUC of 0.54, only marginally better than random classification performance. Although some problematic periods were identified, several production drops were missed, and additional false positive predictions occurred around periods of natural production variability.

Fig. 18 plots the distributions of the RMSE values for the experiment #3 regression problem, demonstrating an effective fit for the regression model with slightly higher performance than that demonstrated in experiment #1 (and much higher performance than that demonstrated in experiment #2). This is also demonstrated in the exemplar production curve in Fig. 19 (representing an overall higher performing flock) which plots the forecast values (red) and target values (blue). We can see that the forecast values follow a very good fit to the target curve, capturing the key features of the curve. The lower RMSE of ∼2.55 indicates higher performance than that demonstrated on the regression task in experiment #1 and #2 (Fig. 8, Fig. 14). This indicates the feature set provides good predictive performance for forecasting the laying rate and that a dataset containing data from both the case study farm and other producers provides the strongest results from the regression task. While high-performing flocks closely mirrored actual production rates, the model's lower-performing scenarios highlighted the limits of single-farm historical training. As seen in the regression task (Fig. 20) for the overall lower performing flock (Flock 7), the model achieved a baseline Root Mean Squared Error of approximately 2.86.

## Discussion

This study demonstrates that machine learning models can effectively forecast egg production drop and laying rate in commercial free-range systems, while also highlighting important limitations in model generalisation across farms. The results reveal a key trade-off between model specificity and generalisability, with important implications for the deployment of predictive tools in the poultry industry.

The findings from Experiment 1 show that data collected within a single farm are sufficient for training machine learning models to forecast production performance in future flocks. When adequate historical data are available, single-farm models can achieve reliable predictive performance, particularly for detecting laying rate fluctuations. This suggests that, in data-rich environments, producers may develop effective forecasting tools without relying on external datasets. However, the benefits of incorporating data from other producers are likely to depend on the similarity between production systems, as well as the quality and consistency of the available data. Free-range egg production systems are inherently heterogeneous, with outcomes influenced by environmental conditions and management practices. As a result, data from one farm may not fully represent conditions in another, potentially limiting model transferability across regions (Prity et al., 2024). These findings are particularly relevant given that data sharing remains a sensitive issue in agriculture, with concerns related to data ownership, privacy, and trust continuing to constrain collaboration (Franzo et al., 2023; Jakku et al., 2019; Wiseman et al., 2019).

The results from Experiment 2 demonstrate that models trained on data from external producers can still provide meaningful predictive performance when applied to a target farm. However, performance differed between regression and classification tasks, with higher accuracy observed for classification. This indicates that models generalise more effectively when identifying abnormal production events than when predicting continuous laying rates. This difference is expected, as classification represents a simpler predictive task that is less sensitive to farm-specific variability, whereas regression requires precise estimation of production values. From a practical perspective, these findings suggest that, in the absence of farm-specific historical data, externally trained models may still provide valuable insights, particularly for detecting production anomalies. This has important implications for smaller producers with limited data infrastructure, who may benefit from models developed using data from larger operations. This highlights the potential value of anonymised data-sharing frameworks to support broader adoption of predictive analytics within the poultry sector. These observations are consistent with previous studies demonstrating that models trained on multi-location datasets can generalise effectively to new production environments (Khaki and Wang, 2019). The results from Experiment 3 further demonstrate that integrating multi-farm datasets with farm-specific historical data can enhance predictive performance, particularly for regression tasks. The combined dataset achieved the lowest RMSE, indicating improved accuracy in forecasting laying rates. This suggests that incorporating diverse datasets enables the model to capture broader production patterns while retaining sensitivity to farm-specific characteristics. These findings align with previous research demonstrating the benefits of aggregating data across multiple sources. For example, Shraga et al. (2022) showed that pooled datasets can improve predictive performance in livestock production systems, even when farm-specific data are limited. Similarly, Coble et al. (2018) and Sharma et al. (2018) highlighted that multi-farm data integration can reveal previously unobserved patterns and enhance model robustness. The approach adopted in this study is also consistent with Liu et al. (2025), who demonstrated that models trained on integrated datasets from multiple pig farms maintained strong performance in previously unseen environments. Collectively, these findings emphasise the potential of multi-farm data integration to improve the scalability and applicability of predictive models in livestock production systems.

Finally, this study highlights the substantial variability in the forecasting performance achieved between different flocks (even those from the same producer) in both regression and classification tasks (Adejola et al., 2025b). This variation likely arises from the differences in the environmental conditions (e.g. temperature, humidity), practises applied to management of the flock, variations in presence of disease and the physical location of the flocks under study across the production period (i.e. differences in the housing) (Gonzalez-Mora et al., 2022). Variation within the predictive performance across the different flocks has reviewed limited attention previous studies and warrants further investigation (Morales et al., 2016; Ramirez-Morales et al., 2017).

## Conclusion

This research presents a detailed case study demonstrating how machine learning can be applied to the production data collected by a free-range egg producer to create a decision support system that forecasts production outcomes (i.e. future laying rate and problematic production days). The case study demonstrates that data collected from previous flocks for a given egg producer can be used to predict future outcomes for that producer. This is a very important finding as it suggests that, where sufficient historical data are available, producers may develop reliable forecasting models without necessarily relying on external datasets. This may allow for the development of decision support platforms that focus on the historical data for a producer rather than a data-sharing approach, which may be a desirable approach given the strong resistance to data sharing across agriculture sector. This finding is important as historical datasets are not always available for the training process, meaning that the use of data from other free-range producers may be the only option. The performance achieved by models trained in this way was comparable to those trained on data from the case study farm alone. Building on this finding, the study demonstrated that a combined dataset that contains data collected from multiple egg producers as well as previous data from the case study farm provided better performance for the regression task and lower performance on the classification task. This indicates that there is no significant benefit from the combined approach. This study has certain limitations that highlight the need for future research. One key limitation is the use of a limited set of features; incorporating a broader range of features in future analyses may enhance the accuracy and reliability of the predictive models. Additionally, the performance of a model developed for one farm may not necessarily translate to others without proper validation, due to variations in management practices and environmental conditions across farms. Overall, this study demonstrates that machine learning models can serve as practical decision-support tools for commercial free-range egg producers by providing early warning of abnormal production events and forecasting future laying performance. When integrated with routine farm monitoring systems, these models may assist producers in improving flock management, responding more rapidly to environmental challenges and reducing production losses through proactive intervention. Further research should incorporate detailed health, vaccination, nutritional, and management-event records to improve biological interpretability and enable the development of cause-specific predictive models.

## Ethics statement

Not applicable. This research used historical production and environmental data previously collected as part of routine farm management activities, and no new data was gathered specifically for this study.

## Funding

We express our gratitude to the Australian Eggs (grant number 31RS103UN) and the Future Food Systems Cooperative Research Centre (grant number P1-017) for their support in funding this project.

## CRediT authorship contribution statement

**Yusuf Adewale Adejola:** Writing – original draft, Visualization, Investigation, Formal analysis, Data curation, Conceptualization. **Terence Zimazile Sibanda:** Writing – review & editing, Methodology, Investigation, Funding acquisition, Data curation, Conceptualization. **Isabelle Ruhnke:** Writing – review & editing, Supervision, Methodology, Funding acquisition, Conceptualization. **Johan Boshoff:** Writing – review & editing, Software. **Mitchell Welch:** Writing – review & editing, Visualization, Supervision, Software, Project administration, Investigation, Funding acquisition, Formal analysis, Data curation, Conceptualization.

## Disclosures

The authors declare that they have no known competing financial interests or personal relationships that could have appeared to influence the work reported in this paper.
